# Limbic-predominant age-related TDP-43 encephalopathy in the oldest old: a population-based study

**DOI:** 10.1093/brain/awae212

**Published:** 2024-06-28

**Authors:** Elizaveta Mikhailenko, Kia Colangelo, Jarno Tuimala, Mia Kero, Sara Savola, Anna Raunio, Eloise H Kok, Maarit Tanskanen, Mira Mäkelä, Henri Puttonen, Mikko I Mäyränpää, Darshan Kumar, Karri Kaivola, Anders Paetau, Pentti J Tienari, Tuomo Polvikoski, Liisa Myllykangas

**Affiliations:** Department of Pathology, University of Helsinki, 00014 Helsinki, Finland; Department of Pathology, University of Helsinki, 00014 Helsinki, Finland; Department of Pathology, University of Helsinki, 00014 Helsinki, Finland; Department of Pathology, University of Helsinki, 00014 Helsinki, Finland; Department of Pathology, HUS Diagnostic Center, Helsinki University Hospital, 00029 Helsinki, Finland; Department of Pathology, University of Helsinki, 00014 Helsinki, Finland; Department of Pathology, HUS Diagnostic Center, Helsinki University Hospital, 00029 Helsinki, Finland; Department of Pathology, University of Helsinki, 00014 Helsinki, Finland; Department of Pathology, HUS Diagnostic Center, Helsinki University Hospital, 00029 Helsinki, Finland; Department of Pathology, University of Helsinki, 00014 Helsinki, Finland; Department of Pathology, University of Helsinki, 00014 Helsinki, Finland; Department of Pathology, University of Helsinki, 00014 Helsinki, Finland; Department of Pathology, University of Helsinki, 00014 Helsinki, Finland; Department of Pathology, HUS Diagnostic Center, Helsinki University Hospital, 00029 Helsinki, Finland; Department of Pathology, University of Helsinki, 00014 Helsinki, Finland; Department of Pathology, HUS Diagnostic Center, Helsinki University Hospital, 00029 Helsinki, Finland; Aiforia Technologies Plc., 00150 Helsinki, Finland; Translational Immunology, Research Programs Unit, University of Helsinki, Helsinki, 00014 Finland; Department of Neurology, University of Helsinki and Helsinki University Hospital, 00014 Helsinki, Finland; Department of Pathology, University of Helsinki, 00014 Helsinki, Finland; Department of Pathology, HUS Diagnostic Center, Helsinki University Hospital, 00029 Helsinki, Finland; Translational Immunology, Research Programs Unit, University of Helsinki, Helsinki, 00014 Finland; Department of Neurology, University of Helsinki and Helsinki University Hospital, 00014 Helsinki, Finland; Translational and Clinical Research Institute, Newcastle University, Newcastle upon Tyne NE1 7RU, UK; Department of Pathology, University of Helsinki, 00014 Helsinki, Finland; Department of Pathology, HUS Diagnostic Center, Helsinki University Hospital, 00029 Helsinki, Finland

**Keywords:** very old, neuropathology, multivariate analysis, autopsy, neurodegeneration, mixed pathology

## Abstract

Population-based cohort studies are essential for understanding the pathological basis of dementia in older populations. Previous studies have shown that limbic-predominant age-related TDP-43 encephalopathy neuropathologic change (LATE-NC) increases with age, but there have been only a few studies, which have investigated this entity in a population-based setting. Here we studied the frequency of LATE-NC and its associations with other brain pathologies and cognition in a population aged ≥ 85 years.

The population-based Vantaa 85+ study cohort includes all 601 individuals aged ≥85 years who were living in Vantaa, Finland in 1991. A neuropathological examination was performed on 304 subjects (50.5%) and LATE-NC staging was possible in 295 of those. Dementia status and Mini-Mental State Examination (MMSE) scores were defined in the baseline study and three follow-ups (1994–99). The LATE-NC stages were determined based on TDP-43 immunohistochemistry, according to recently updated recommendations. Arteriolosclerosis was digitally assessed by calculating the average sclerotic index of five random small arterioles in amygdala and hippocampal regions, and frontal white matter. The association of LATE-NC with arteriolosclerosis and previously determined neuropathological variables including Alzheimer’s disease neuropathologic change (ADNC), Lewy-related pathology (LRP), hippocampal sclerosis (HS) and cerebral amyloid angiopathy (CAA), and cognitive variables were analysed by Fisher’s exact test, linear and logistic regression (univariate and multivariate) models.

LATE-NC was found in 189 of 295 subjects (64.1%). Stage 2 was the most common (28.5%) and stage 3 the second most common (12.9%), whereas stages 1a, 1b and 1c were less common (9.5%, 5.1% and 8.1%, respectively). Stages 1a (*P* < 0.01), 2 (*P* < 0.001) and 3 (*P* < 0.001) were significantly associated with dementia and lower MMSE scores. LATE-NC was associated with ADNC (*P* < 0.001), HS (*P* < 0.001), diffuse neocortical LRP (*P* < 0.002), and arteriolosclerosis in amygdala (*P* < 0.02). In most cases LATE-NC occurred in combination alongside other neuropathological changes. There were only six subjects with dementia who had LATE-NC without high levels of ADNC or LRP (2% of the cohort, 3% of the cases with dementia), and five of these had HS. In all multivariate models, LATE-NC was among the strongest independent predictors of dementia. When LATE-NC and ADNC were assessed in a multivariate model without other dementia-associated pathologies, the attributable risk was higher for LATE-NC than ADNC (24.2% versus 18.6%).

This population-based study provides evidence that LATE-NC is very common and one of the most significant determinants of dementia in the general late-life aged population.

## Teaser

Mikhailenko *et al.* examine the frequency of limbic-predominant age-related TDP-43 encephalopathy (LATE-NC) in a population aged ≥85 years, and conclude that it is very common—affecting more than 50% of individuals—and one of the most significant determinants of dementia in the general oldest-old population.

## Introduction

The human population is experiencing a demographic shift, with a growing number of individuals reaching 80 years of age and older, posing significant challenges in terms of age-related neurodegenerative diseases.^1^ First, the number of people with dementia is going to expand as the prevalence of dementia is highest in the oldest age groups. Second, recent studies have revealed that novel disease entities such as limbic-predominant age-related TDP-43 encephalopathy neuropathologic change (LATE-NC) and primary age-related tauopathy (PART) are particularly common among the oldest old.^1-4^ Thus, with increasing numbers of individuals aged 80+, the proportion of subjects with these novel entities will substantially increase in the future. Third, population-based studies have shown that late-life dementia often involves a complex interplay of different brain pathologies. The impact of mixed pathologies, in particular those involving novel entities such as LATE-NC, on dementia within elderly populations is still incompletely understood. There is a limited number of population-representative studies focused on the oldest old age groups, where the impact of novel entities in combination with other diseases can be studied in an unbiased manner.^4-12^

LATE-NC is characterized by abnormal accumulation of TAR DNA-binding protein 43 (TDP-43) mainly in the amygdala and hippocampus, and to some extent also in cortical areas in older individuals. Currently, LATE-NC can only be definitively diagnosed at autopsy.^13^ Although TDP-43 pathology had been recognized as an independent factor influencing cognition in earlier studies,^14^ the term LATE-NC was introduced in the consensus article by Nelson *et al*.^3^ in 2019 which, in addition to defining nomenclature, presented recommendations for the neuropathological staging system (stages 1–3) of LATE-NC. These recommendations were recently updated in 2023, by adding more specificity regarding the neuroanatomical regions studied and introducing some novel stages specifying the type of pathology present (stages 1a, 1b and 1c), with the aim of clarifying the proper use of the LATE-NC staging system and to increase reproducibility between studies.^13^

Although age-associated TDP-43 accumulation in the brain is known to be common and associated with memory loss, a definition and description of LATE-NC is still debated.^15,16^ Comprehensive studies investigating LATE-NC prevalence, clinical correlations, and relationships with other common neuropathologies, including Alzheimer’s disease neuropathologic change (ADNC), hippocampal sclerosis (HS), Lewy body disease (LBD) and vascular changes, remain limited. In particular, the boundaries between LATE-NC and ADNC, HS or LBD are obscure, as LATE-NC is often seen in combination with at least one of these entities. Furthermore, it is still unclear if LATE-NC alone can explain dementia, and if so, how common such a dementia is.

Here we assessed the frequency of different stages of LATE-NC according to the updated neuropathological recommendations,^13^ and their associations with cognitive parameters and other brain pathologies, in the unique, prospectively examined, population-based Vantaa 85+ cohort. This population cohort included all individuals aged ≥85 years who had been living in the city of Vantaa, Finland, on 1 April 1991. The subsequent autopsy frequency reached >50%. Thus, this cohort is an excellent resource for studying the impact of LATE-NC, a disorder particularly common among the oldest old.^1,3,17^ The specific aim of the study was to elucidate the impact of LATE-NC, in combination with other pathologies, on dementia in an unselected very elderly population.

## Materials and methods

### Subjects

The Vantaa 85+ study consists of all individuals aged 85 years or older who were residents of the city of Vantaa as of 1 April 1991 (*n* = 601). Supplementary Fig. 1 presents a flow chart of the participants of the Vantaa 85+ Study. Clinical examinations were conducted on 553 patients. Autopsy and neuropathological examination was possible for 304 (50.5%) subjects. The demographic details of the whole Vantaa 85+ study sample (*n* = 601) and the neuropathologically examined subsample (*n* = 304) are shown in Supplementary Table 1. An exact description of the Vantaa 85+ study has been described before.^18-20^

Blood samples for genetic testing and lipid analyses were collected from 553 patients. The baseline clinical study was conducted in 1991 and 1992, with follow-up assessments carried out periodically until 2001. Dementia status was determined using The Diagnostic and Statistical Manual of Mental Disorders (DSM-III-R) criteria and Mini-Mental State Examination (MMSE) scores were evaluated both at the study’s inception and during subsequent follow-up assessments in 1994, 1996 and 1999.^20^ The methods for apolipoprotein E (*APOE*) genotyping have been described previously.^21^ Genetic data for transmembrane protein 106B (*TMEM106B)* and granulin (*GRN)* variants were determined from imputed whole-genome genotyping data described previously.^22^

The ethical committee of the city of Vantaa and the Helsinki University Hospital have approved the study. The Finnish Health and Social Ministry permitted the assessment of death certificates and health and social work records for research. Collection of the tissue samples and their research use was approved by the National Authority for Medicolegal Affairs (VALVIRA). Each participant and/or their relatives have given informed consent for the study.

### Histological analyses

After consented post-mortem examination brains were fixed in formalin for at least 2 weeks before sampling. Samples from the right hippocampus at the coronal level of the lateral geniculate body, amygdala at the level of uncus, and middle frontal gyrus were used for this study. The PEG-embedded hippocampal and amygdala samples were later re-embedded in paraffin as described by Kero *et al*.,^23^ whereas the frontal sample was already initially paraffin-embedded. A rabbit polyclonal anti-C-terminal-TDP-43 antibody (Proteintech, concentration 0.3 mg/ml) was used with LabVision immunostainers and the Agilent Dako EnVision FLEX+ detection system (K8002 kit with rabbit linker K8009). Prior to staining, tissue sections of 4 μm underwent epitope retrieval pre-treatment in a BioCare Medical Decloaking Chamber™ NxGen for 20 min at 95°C with DAKO K8005 kit citrate buffer at pH 6.1 and formic acid treatment for 10 min at room temperature.

Presence of TDP-43 positive neurofibrillary tangle-like and granular neuronal cytoplasmic inclusions (NCI) and TDP-43 positive processes were evaluated in each section, according to the updated criteria by Nelson *et al*.^13^ (Supplementary Fig. 2). Briefly, stage 1a represented cases with TDP-43 positive NCI only in the amygdala. Stage 1b represented cases with TDP-43 positive NCIs only in the hippocampus. For stage 1c, which includes cases with TDP-43-positive cell processes but without NCI in amygdala and/or hippocampus, we accepted cases that showed at least three TDP-43 positive processes in either amygdala and/or hippocampus in the absence of NCI. The requirement of at least three processes was used to increase the likelihood that this minor pathology represented real TDP-43 pathology and not artefactual staining that is sometimes seen, for example, at the pial lining. Stage 2 included cases who had TDP-43 positive NCI in both amygdala and hippocampal sections, and stage 3 cases had TDP-43 positive NCI in all three investigated brain regions.^13^

To assess arteriolosclerosis, we used digitalized methods and the Aiforia software (Aiforia Technologies Plc., Helsinki, Finland) to determine the sclerotic index (SI) of five random arterioles in the same samples of frontal lobe, amygdala and hippocampus that were immunohistochemically stained for TDP-43.^24,25^ First, 4-µm thick sections were stained with haematoxylin and eosin and scanned with a 3D HISTECH Panoramic 250 Flash III scanner using bright field at ×200 magnification. Scanned slides were then uploaded to the Aiforia cloud service for training. Algorithms were first trained to recognize tissue, separate blood vessels from background tissue, and in the frontal sections to recognize vessels in the white matter. We included only vessels with a diameter of 20–150 µm, as described previously by Skrobot *et al*.^26^ and cut perpendicularly, so that the vessel wall, as well as the possible hyaline layer were clearly measurable. The algorithms for all three anatomical areas were validated by three pathologists to ensure the quality of the algorithm before the final run. After the final run, each slide was investigated manually, and five random vessels were chosen for further analyses. For each chosen vessel, the outer and inner diameter were measured from two separate angles (Supplementary Fig. 2).^24,25^ These measurements were used to calculate the SI for each chosen vessel using the following formula: SI = 1 − (Inner diameter 1 + Inner diameter 2) / (Outer diameter 1 + Outer diameter 2). The average SI of the five vessels was used in the final analyses.

The methodological details for other brain pathologies have been described elsewhere: CERAD score,^20^ Modified Braak staging and Thal phase assessment, PART, and argyrophilic grain disease (AGD),^22^ Lewy-related pathology (LRP) associated with LBD,^27^ HS and circle of Willis atherosclerosis,^23^ CAA,^28^ brain infarcts,^29^ microhaemorrhages and -infarcts.^19^ The ADNC score was categorized using the Braak, Thal and CERAD assessments according to Montine *et al*.^30^ and Hyman *et al*.^31^

### Statistical analyses

Fisher’s exact test was used to test for associations between categorical variables in Tables 1 and 2. Linear regression models were used to estimate the effects of explanatory variables on continuous outcomes in Tables 1, 2 and 4– 6. Logistic regression was applied to binary dependent variables in Tables 2 and 6. Multivariate logistic regression was used to model the effect of various predictive variables on dementia (Table 7). We fitted several models with different combinations of brain pathologies to study the possible combined effect on the presence or absence of dementia. Results of multivariate logistic regression models were also used to estimate the fraction of dementia cases attributable to each dementia-associated pathology (attributable risk). In addition, multivariate regression models were used for adjustments for ADNC and/or LRP pathologies in Tables 1 and 2 and for age at death and sex in Tables 2–7. The genetic variables *GRN rs5848* and *TMEM106B rs1990622* were analysed using the R v.4.1.2 stats package with Fisher’s exact test using an allelic model. The *P*-value was approximated using Monte Carlo simulation with 10 000 replicates. Attributable fractions were analysed using R v.4.1.2 with the package AF.^32,33^ All other statistical analyses were carried out using SPSS version 28. Adjustments for multiple correction were not applied. *P* < 0.05 was considered significant.

**Table 1:** Characteristics of subjects with and without LATE-NC

	All^a^	LATE-NC Stage						*P* ^b^
		0	1a	1b	1c	2	3	
*n* (%)	295	106 (35.9)	28 (9.5)	15 (5.1)	24 (8.1)	84 (28.5)	38 (12.9)	
Sex, male/female	51/244	16/90	7/21	5/10	7/17	12/72	4/34	NS
Age at death, years, median	92.1	91.22	90.67	92.95	91.35	92.85	93.21	NS
Smokers^c^, yes, *n* (%)	36 (12.2)	16 (15.1)	3 (10.7)	2 (13.3)	6 (25.0)	6 (7.1)	3 (7.9)	NS
Total cholesterol^d^, mmol/l, median	5.4	5.3	5.0	6.0	5.7	5.4	5.2	NS
LDL^e^, mmol/l, median	3.5	3.30	3.2	3.8	3.7	3.6	3.6	NS
HDL^d^, mmol/l, median	1.0	0.98	0.9	1.0	0.9	1.0	1.0	NS
TGL^d^, mmol/l, median	1.6	1.65	1.9	1.6	2.0	1.58	1.51	0.092
Hypertension medication^f^, yes, *n* (%)	131 (44.4)	53 (50.0)	17 (60.7)	7 (46.7)	11 (45.8)	30 (35.7)	13 (34.2)	0.089
DM2 medication, *n* (%)	62 (21.0)	26 (24.5)	6 (21.4)	3 (20.0)	6 (25.0)	17 (20.2)	4 (10.5)	NS
*APOE ε4* ^g^, *n* (%)	88 (29.8)	21 (19.8)	13 (46.4)	6 (40.0)	5 (20.8)	33 (39.3)	10 (26.3)	0.015 (NS)^h^
*GRN rs5848^i^*								**<0.001**
C-allele	357	142 (78.0)	38 (70.4)	19 (67.9)	35 (76.1)	94 (63.5)	29 (43.9)	
T-allele	167	40 (22.0)	16 (29.6)	9 (32.1)	11 (23.9)	54 (36.5)	37 (56.1)	
*TMEM106B rs1990622^i^*								
A-allele	349	120 (65.9)	33 (61.1)	22 (78.6)	25 (54.3)	96 (64.9)	53 (80.3)	**0.036**
G-allele	175	62 (34.1)	21 (38.9)	6 (21.4)	21 (45.7)	52 (35.1)	13 (19.7)	–

*GRN rs5848* and *TMEM106B rs1990622* were analysed using Fisher’s exact test using an allelic model. Results of association analyses with different variables are not adjusted for age and sex; confidence interval 95%. *APOE ε4* = apolipoprotein E *ε4* allele; DM2 = type 2 diabetes mellitus; *GRN* = granulin; HDL = high-density lipoprotein; LDL = low-density lipoprotein; LATE-NC = limbic-predominant age-related TDP-43 encephalopathy neuropathologic change; NS = no statistical significance; TGL = triglycerides; *TMEM106B* = transmembrane protein 106B. Significant *P*-values (*P* < 0.05) are in bold.

^a^Number of neuropathologically examined subjects with LATE-NC staging possible.

^b^Fisher’s exact test analysing all stages together for dichotomous variables, linear regression analysis for continuous variables.

^c^Smoker status known for 248/295 participants.

^d^Total cholesterol, HDL cholesterol and triglyceride values available for 258/295 participants.

^e^LDL data available for 242/295 subjects.

^f^Data on hypertension medication available for 283/295 participants.

^g^APOE *ε4* status available for 275/295 participants.

^h^When adjusted with ADNC, *P* = 0.65.

^i^Data available for 262/295 participants.

**Table 2:** Results of association analyses between LATE-NC and cognitive parameters

	All^a^	LATE-NC Stage						*P*
		0	1a	1b	1c	2	3	
*n* (%)	295	106 (35.9)	28 (9.5)	15 (5.1)	24 (8.1)	84 (28.5)	38 (12.9)	–
Age at death, years, median	92.1	91.2	90.7	93.0	91.4	92.9	93.2	NS
Dementia, yes, *n* (%)	189 (64.1)	49 (46.2)	21 (75.0)	10 (66.7)	10 (41.7)	66 (78.6)	33 (86.8)	**<0.001**
Age of dementia onset^b^, years, median	87.3	87.2	87.9	86.8	89.8	85.6	86.4	NS
Duration of dementia^c^, years, median	4.4	4.1	3.4	4.9	1.6	5.3	6.6	NS
MMSE at baseline 1991^d^, median	18	21	16	19	24	13	11.5	NS
Last MMSE score baseline included^e^, median	13.5	18	14	14	21	8	7	NS

LATE-NC Stage	OR (*P*)	Unstandardized B-value (*P*)			
	Dementia (yes/no)	Onset of dementia	Duration of dementia	MMSE at baseline 1991	The last MMSE score baseline included
1a	**3.5 (0.01^f^)**	NS	NS	**−5.3 (0.01^f^)**	**−5.1 (0.01^f^)**
1b	NS	NS	NS	NS	NS
1c	NS	NS	NS	NS	NS
2	**4.5 (<0.001^h^)**	**−2.1 (0.001^g^)**	**2.3 (<0.001^g^)**	**−6.6 (<0.001^h^)**	**−7.5 (<0.001^h^)**
3	**7.8 (<0.001^h^)**	**−3.1 (<0.001^g^)**	**3.4 (<0.001^h^)**	**−7.5 (<0.001^h^)**	**−8.7 (<0.001^h^)**

*Top*: All stages analysed together (Fisher’s exact test). Results of association analyses with different variables are not adjusted for age and sex; confidence interval 95%. *Bottom*: Individual stages separately compared with LATE -NC stage 0 (regression analyses). Results of multivariate logistic regression analyses with the dichotomous variable (dementia) and multivariate linear regression for the continuous variables adjusted for sex and age. Significant *P*-values (*P* < 0.05) in bold. LATE-NC = limbic-predominant age-related TDP-43 encephalopathy neuropathologic change; MMSE = Mini-Mental State Examination; NS = non-significant (*P* > 0.05).

^a^Number of neuropathologically examined subjects with LATE-NC staging possible.

^b^Data on the onset of dementia available for 188/189 participants with dementia.

^c^Data on the duration of dementia available for 185/189 participants with dementia.

^d^Median MMSE scores at baseline (1991) for each group out of a maximum of 30 points, MMSE score data available at baseline for 278 participants.

^e^The last MMSE score, baseline included, data available for 278 participants.

^f^Significant (*P* < 0.05) after adjustment for ADNC and LRP.

^g^Significant (*P* < 0.01) after adjustment for ADNC and LRP.

^h^Significant (*P* < 0.001) after adjustment for ADNC and LRP.

**Table 3:** Association analyses of neurodegenerative pathologies in subjects with and without LATE-NC

		LATE-NC Stage				
Variable	All^a^ n = 295	0 n = 106	1a n = 28	2 n = 84	3 n = 38	*P^b^*
CERAD score^c^, *n* (%)						
None	67 (22.7)	31 (29.2)	6 (21.4)	15 (17.9)	6 (15.8)	Ref.
Sparse	32 (10.8)	15 (14.2)	1 (3.6)	6 (7.1)	6 (15.8)	NS
Moderate	158 (53.6)	52 (49.1)	17 (60.7)	46 (54.8)	19 (50.0)	**0.050**
Frequent	38 (12.9)	8 (7.5)	4 (14.3)	17 (20.2)	7 (18.4)	**0.003**
Braak NFT stage^d^, *n* (%)						
0–II	52 (17.6)	31 (29.2)	1 (3.6)	6 (7.1)	7 (18.4)	Ref.
III–IV	140 (47.5)	54 (50.9)	13 (46.4)	32 (38.1)	19 (50.0)	**0.015**
V–VI	102 (34.6)	20 (18.9)	14 (50.0)	46 (54.8)	12 (31.6)	**<0.001**
Thal phase^e^, *n* (%)						
0	17 (5.8)	10 (9.4)	2 (7.1)	1 (1.2)	0	Ref.
1–2	45 (15.3)	23 (21.7)	2 (7.1)	10 (11.9)	6 (15.8)	NS
3	27 (9.1)	11 (10.4)	2 (7.1)	8 (9.5)	2 (5.3)	NS
4–5	206 (69.8)	62 (58.5)	22 (78.7)	65 (77.4)	30 (78.9)	**0.010**
ADNC score^f^, *n* (%)						
No–low	93 (31.5)	51 (48.1)	5 (17.9)	15 (17.9)	10 (26.3)	Ref.
Intermediate	102 (34.6)	34 (32.1)	9 (32.1)	24 (28.6)	16 (42.1)	**0.007**
High	99 (33.6)	20 (18.9)	14 (50.0)	45 (53.5)	12 (31.6)	**<0.001**
LRP^g^, *n* (%)						
None	175 (59.3)	72 (67.9)	15 (53,6)	46 (54.8)	20 (52.6)	Ref.
Brainstem predominant	19 (6.4)	7 (6.6)	0	4 (4.8)	5 (13.2)	NS
Limbic predominant	39 (13.2)	16 (15.1)	3 (10.7)	11 (13.1)	4 (10.5)	NS
Diffuse neocortical	41 (13.9)	6 (5.7)	5 (17.9)	18 (21.4)	7 (18.4)	**0.002**
Amygdala predominant	10 (3.4)	1 (0.9)	3 (10.7)	3 (3.6)	0	NS
AGD^h^, *n* (%)	80 (27.1)	27 (25.5)	8 (28.6)	28 (33.3)	7 (18.4)	NS
PART^i^, *n* (%)	57 (19.3)	31 (29.2)	4 (14.3)	10 (11.9)	5 (13.2)	**<0.001^j^**
HS^k^, *n* (%)	46 (15.6)	2 (1.9)	1 (3.6)	18 (21.4)	25 (65.8)	**<0.001**

Significant *P*-values (*P* < 0.05) are in bold. AGD = argyrophilic grain disease; CERAD = Consortium to Establish a Registry for Alzheimer’s Disease; HS = hippocampal sclerosis; LATE-NC = limbic-predominant age-related TDP-43 encephalopathy neuropathologic change; LRP = Lewy-related pathology; ADNC = Alzheimer’s disease neuropathologic change; NFT = neurofibrillary tangle; NS = no statistical significance; PART = primary age-related tauopathy; Ref. = reference.

^a^Number of neuropathologically examined subjects with LATE-NC staging possible, includes subjects with LATE-NC stage 1b (*n* = 15) and 1c (*n* = 24).

^b^Stages 1a + 2 + 3 versus 0 compared using logistic regression analysis, adjusted for age at death and sex.

^c^Neuropathological protocol for scoring neuritic plaques, data published previously.^20^

^d^Modified Braak staging protocol for Tau-pathology assessment. Data available for 294/295 participants.^22^

^e^Staging protocol for Aβ-deposits by Thal *et al*.^22,34^

^f^Data available for 294/295 participants.

^g^Lewy-related pathology. Non-classifiable class (*n* = 11) excluded. Dementia with Lewy Bodies (DLB) Consortium classification for LRP, data published previously.^27^

^h^Argyrophilic grain disease, data available for 294/295.^22^

^i^Primary age-related tauopathy defined as Braak Stages 1–4 and Thal Stages 0–2 and available for 292/295 participants, data published previously.^22^

^j^Inverse association.

^k^Hippocampal sclerosis, data published previously.^23^

**Table 4:** Association analyses of vascular pathologies in subjects with and without LATE-NC

	LATE-NC Stage					
	All^a^ *n* = 295	0 *n* = 106	1a *n* = 28	2 *n* = 84	3 *n* = 38	*P^b^*
Cerebral infarct^c^, *n* (%)						
Small cortical	57 (19.3)	16 (15.1)	8 (28.6)	17 (20.2)	10 (26.3)	NS
Large cortical	51 (17.3)	23 (21.7)	6 (21.4)	8 (9.5)	5 (13.2)	0.089
Small white matter	44 (14.9)	10 (9.4)	8 (28.6)	15 (17.9)	8 (21.1)	**0.022**
Large white matter	6 (2.0)	2 (1.9)	1 (3.6)	2 (2.4)	1 (2.6)	NS
Small basal ganglia	59 (20.0)	17 (16.0)	5 (17.9)	19 (22.6)	11 (28.9)	NS
Small brainstem	13 (4.4)	2 (1.9)	2 (7.1)	4 (4.8)	3 (7.9)	NS
Small cerebellum	53 (18.0)	13 (12.3)	6 (21.4)	21 (25.0)	7 (18.4)	**0.043**
Large cerebellum	15 (5.1)	3 (2.8)	2 (7.1)	6 (7.1)	2 (5.3)	NS
Anterior circulation	120 (40.7)	40 (37.7)	14 (50.0)	37 (44.0)	16 (42.1)	NS
Posterior circulation	98 (33.2)	26 (24.5)	12 (42.9)	32 (38.1)	16 (42.1)	**0.011**
CAA^d^, *n* (%)						
No CAA	76 (25.8)	35 (33.0)	6 (21.4)	15 (17.9)	7 (18.4)	Ref.
CAA - type 1	83 (28.1)	20 (18.9)	14 (50.0)	26 (30.9)	13 (34.2)	<0.001 (NS)^e^
CAA - type 2	132 (44.7)	48 (45.3)	8 (28.6)	43 (51.2)	18 (47.4)	0.059
Arteriolosclerosis (SI)^f^, median						
Amygdala	0.36	0.34	0.35	0.37	0.38	**0.011**
Hippocampus	0.37	0.36	0.34	0.37	0.39	NS
Frontal white matter	0.38	0.37	0.37	0.40	0.41	**0.015**
Cortical microhaemorrhages^g^, *n* (%)	180 (61.0)	65 (61.3)	16 (57.1)	51 (60.7)	24 (63.2)	NS
Microinfarcts^g^, *n* (%)	49 (16.6)	13 (12.3)	5 (17.9)	19 (22.6)	7 (18.4)	NS
Moderate or severe brain atherosclerosis at the circle of Willis^h^, *n* (%)	181 (61.4)	67 (63.2)	12 (42.9)	56 (66.7)	27 (71.1)	NS

Significant *P*-values (*P* < 0.05) are in bold. CAA = cerebral amyloid angiopathy; LATE-NC = limbic-predominant age-related TDP-43 encephalopathy neuropathologic change; NS = no statistical significance; SI = Sclerotic Index; Ref. = reference.

^a^Number of neuropathologically examined subjects with LATE-NC staging possible, includes subjects with LATE-NC stage 1b (*n* = 15) and 1c (*n* = 24).

^b^Stages 1a + 2 + 3 versus 0 compared using logistic regression for dichotomous variables, linear regression analysis for continuous variables, adjusted for age at death and sex.

^c^Data published previously.^29^

^d^Cerebral amyloid angiopathy, data available for 291/295. Data published previously.^28^

^e^When adjusted with ADNC, *P* < 0.66.

^f^Sclerotic Index (SI).^24^ Data on amygdala available for 288/295, hippocampus for 294/295 and frontal white matter for 277/295.

^g^Data on microinfarcts and cortical microhaemorrhages available for 291/295.^19^

^h^Data available for 275/295, subjects with moderate or severe compared with no or mild atherosclerosis.^23^

**Table 5:** Clinical and neuropathological characteristics of subjects with different combinations of LATE-NC, ADNC and LRP

	ADNC only^a^ *n* = 33	ADNC + LATE-NC^b^ *n* = 61	ADNC + LATE-NC + LRP^c^ *n* = 48	LATE-NC only^d^ *n* = 20
Dementia, yes, *n* (%)	18 (54.5)	49 (80.3)	45 (93.8)**	12 (60.0)
Sex, male/female	5/28	8/53	9/39	2/18
Age at death, median	91.5	92.3	91.9	92.8
Age at onset, median	86.8	86.9^#^	86.3*	89.3
Duration of dementia, median	4.4	4.9^#^	6.3*	2.5
MMSE score at baseline 1991, median	21	13.5^#^	8.5**	21
Last MMSE score baseline included, median	18	8^##^	0***	14
LATE-NC Stage, *n* (%)				
1a	0	11 (18.0)	10 (20.8)	4 (20.0)
2	0	35 (57.4)	29 (60.4)	11 (55.0)
3	0	15 (24.6)	9 (18.8)	5 (25.0)
HS, *n* (%)	0	21 (34.4)	13 (27.1)	6 (30.0)
CAA, *n* (%)	29 (87.9)	53 (86.9)^###^	45 (93.8)***	8 (40.0)
PART, *n* (%)	0	0	0	11 (55.0)
Arteriolosclerosis (SI), median				
Amygdala	0.34	0.36	0.38	0.35
Hippocampus	0.37	0.38	0.38	0.35
Frontal white matter	0.39	0.40^#^	0.40	0.34

Associations between groups LATE-NC + ADNC + LRP and LATE-NC only, or ADNC+ LATE-NC only, are determined using sex and age-adjusted linear regression for continuous variables and logistic regression for the dichotomous dementia variable. When comparing LATE-NC + ADNC + LRP versus LATE-NC only: *P < 0.05; **P < 0.01; ***P < 0.001. When comparing ADNC + LATE-NC versus LATE-NC only: ^#^*P* < 0.05; ^##^*P* < 0.01; ^###^*P* < 0.001. ADNC = Alzheimer’s disease neuropathologic change; CAA = Cerebral amyloid angiopathy; HS = hippocampal sclerosis; LATE-NC = limbic-predominant age-related TDP-43 encephalopathy neuropathologic change; LRP = Lewy related pathology; MMSE = Mini-Mental State Examination; NS = no statistical significance.

^a^ADNC only defined as presence of ADNC intermediate or high,^30^ but no LATE-NC stage 1a, 2 or 3 and no LRP (non-classifiable subgroup excluded).

^b^ADNC + LATE-NC group defined as presence of ADNC intermediate or high^30^ with LATE-NC stage 1a, 2 or 3 and no LRP (non-classifiable subgroup excluded).

^c^ADNC + LATE-NC + LRP group defined as presence of ADNC intermediate or high^30^ with LATE-NC stage 1a, 2 or 3 and LRP (non-classifiable and brainstem-predominant subgroups excluded).

^d^LATE-NC only defined as presence of LATE-NC stage 1a, 2 or 3 and no ADNC high or intermediate and no LRP.

**Table 6:** Results of univariate analyses between neuropathological variables and dementia

	Neuropathologically examined population *n* = 304		Dementia
Variable	Demented *n* = 197	Non-demented *n* = 107	OR (95% CI)
ADNC (intermediate or high), *n* (%)	151 (76.6)	53 (49.5)	**3.5** (2.1–5.8)***
LATE-NC (stages 1a or 2 or 3), *n* (%)	120 (60.9)	30 (28.0)	**4.8** (2.7–8.3)***
LRP^a^ of any type, *n* (%)	85 (43.1)	28 (26.2)	**2.2** (1.3–3.6)**
AGD, *n* (%)	52 (26.4)	30 (28.0)	0.9 (0.5–1.6)
PART, *n* (%)	28 (14.2)	31 (29.0)	**0.4** (0.2–0.7)**
HS, *n* (%)	45 (22.8)	2 (1.9)	**15.3** (3.6–64.8)***
Cerebral infarct^b^, *n* (%)			
Small cortical	45 (22.8)	12 (11.2)	**2.4** (1.2–4.7)*
Anterior circulation	87 (44.2)	35 (32.7)	1.6 (1.0–2.6)
Posterior circulation	73 (37.1)	25 (23.4)	**1.9** (1.1–3.2)*
CAA, *n* (%)			
CAA types 1 and 2	156 (79.2)	65 (59.6)	**2.7** (1.6–4.6)***
Arteriolosclerosis (SI), median			
Amygdala	0.36	0.34	**Group effect^c^*P* 0.002** **Group 1: 1.9 (1.0–3.6)** Group 2: 2.6 (1.3–5.3)** Group 3: 3.7 (1.8–7.8)***
Hippocampus	0.37	0.36	Group effect *P* NS Group 1: 0.7 (0.4–1.3) Group 2: 1.3 (0.7–2.7) Group 3: 1.3 (0.7–2.7)
Frontal white matter	0.39	0.36	Group effect *P* NS Group 1: 1.2 (0.6–2.4) Group 2: 1.6 (0.8–3.3) Group 3: 1.9 (0.9–3.8)
Cortical microhaemorrhages, *n* (%)	116 (58.9)	70 (65.4)	0.7 (0.4–1.2)
Microinfarcts, *n* (%)	34 (17.3)	16 (15.0)	1.4 (0.6–2.2)
Moderate or severe brain atherosclerosis in the circle of Willis, *n* (%)	125 (63.5)	63 (58.9)	1.0 (0.6–1.8)

Results of univariable logistic regression analyses with different variables, adjusted for age and sex are shown. Significant *P*-values (*P* < 0.05) are in bold. *P < 0.05, **P < 0.01, ***P < 0.001. ADNC = Alzheimer’s disease neuropathologic change; AGD = argyrophilic grain disease; CAA = cerebral amyloid angiopathy; CERAD = Consortium to Establish a Registry for Alzheimer’s Disease; CI = confidence interval; HS = hippocampal sclerosis; LATE-NC = limbic predominant age-related TDP-43 encephalopathy neuropathologic change; LRP = Lewy-related pathology; NS = no statistical significance; OR = odds ratio; PART = primary age-related tauopathy; SI = Sclerotic Index.

^a^The non-classifiable class of LRP (*n* = 11) is not included in the analyses.

^b^Only significant (*P* < 0.05) or borderline (*P* < 0.1) results are presented.

^c^Statistical analyses conducted with the categorical percentile group variables (quartiles) of arteriolosclerosis for each brain region using logistic regression.

**Table 7:** Multivariate models assessing dementia determinants in the oldest-old population

	Model 1	Model 2	Model 3	Model 4	Model 5	Model 6
Variable	OR (CI 95%) AF					
Sex	1.4 (0.7–3.1)	1.5 (0.7–3.3)	1.3 (0.6–2.9)	1.2 (0.5–2.8)	1.3 (0.6–3.1)	1.0 (0.4–2.4)
Age at death	1.0 (0.9–1.1)	1.0 (0.9–1.1)	1.0 (0.9–1.1)	1.0 (0.9–1.1)	1.0 (0.9–1.1)	1.0 (0.9–1.0)
LATE-NC^a^	**3.4** (1.9–6.0)*** **20.0%**	**3.8** (2.1–6.7)*** **24.2%**	**3.7** (2.0–6.7)*** **23.0%**	**2.5** (1.4–4.8)** **13.7%**	**2.3** (1.2–4.4)** **12.4%**	**2.7** (1.4–5.2)** **13.8%**
ADNC^b^	NI	**2.5** (1.4–4.5)** **18.6%**	**2.6** (1.4–4.7)** **18.6%**	**2.6** (1.4–4.8)** **17.4%**	**2.5** (1.2–4.9)* **15.9%**	**2.5** (1.3–4.8)** **15.8%**
LRP^c^	NI	NI	1.7 (0.9–3.1) 5.0%	1.8 (0.9–3.4) 5.6%	**1.9** (1.0–3.7)* **6.1%**	1.9 (0.9–3.6) 5.1%
HS	**8.6** (2.0–37.6)** **6.3%**	NI	NI	**8.2** (1.8–37.2)** **6.0%**	**8.5** (1.9–38.8)** **6.1%**	**7.4** (1.6–34.3)** **5.4%**
CAA^d^	NI	NI	NI	NI	1.23 (0.6–2.8) 4.9%	NI
SI amygdala^e^	NI	NI	NI	NI	NI	Group effect: NS Group 1: 1.9 (0.8–4.4) Group 2: 2.5 (1.0–6.1)* Group 3: 2.7 (1.1–6.6)* 7.7%

Dementia status was the dependent variable for all models, analyses shown here are conducted with logistic regression. The attributable fraction was calculated for categorical independent variables (age at death and sex included only as control variables). Significant *P*-values (*P* < 0.05) are in bold. *P < 0.05, **P ≤ 0.01, ***P < 0.001. ADNC = Alzheimer’s disease neuropathologic change; AF = Attributable fraction; CAA = cerebral amyloid angiopathy; CI = confidence interval; HS = hippocampal sclerosis; LATE-NC = limbic predominant age-related TDP-43 encephalopathy neuropathologic change; LRP = Lewy-related pathology; NI = not included; NS = not significant; OR = odds ratio; SI = Sclerotic Index.

^a^LATE-NC is a binary variable, and includes stages 1a, 2 and 3 versus stage 0.

^b^ADNC is a binary variable with no-low versus intermediate-high groups.

^c^LRP of any type versus no LRP, the non-classifiable class of LRP (*n* = 11) is not included in the analyses.

^d^Types 1 and 2 of CAA versus no CAA.

^e^Arteriolosclerosis in amygdala, SI average of five random vessels, analysed as a categorical variable (quartiles).

## Results

### Frequencies of LATE-NC stages and demographic and clinical characteristics

Table 1 presents the frequencies of the different LATE-NC stages according to the updated criteria^13^ and their associations with various demographic, clinical and genetic variables. LATE-NC was present in 189 of 295 subjects where LATE-NC staging was possible (64.1%). Stage 2 was the most common stage (found in 28.5% of the study subjects) and stage 3 the second most common (found in 12.9%). Stages 1a (inclusions in amygdala only), 1b (inclusions in hippocampus only) and 1c (processes only in hippocampus and/or amygdala) were less common (9.5%, 5.1% and 8.1%, respectively). In the Vantaa 85+ cohort, no cases were considered consistent with the diagnosis of FTLD-TDP based on neuropathological, clinical or genetic findings (details shown in the Supplementary material).

LATE-NC stages were not associated with age at death and sex. Smoking, blood lipid levels or use of hypertension or diabetes medication were also not significantly associated with LATE-NC stages. Carriership of the apolipoprotein ɛ4 (*APOE4*) allele showed a weak association (*P* < 0.015) with LATE-NC (Table 1), but this association was not significant when adjusted for ADNC pathology and is therefore likely driven by concomitant ADNC pathology in the LATE-NC cases. We also tested for an association between LATE-NC and two genetic variants that have previously been shown to be associated with HS in the Vantaa 85+ study: The *GRN rs5848* and *TMEM106B rs1990622*.^12^ In these analyses, the *GRN* variant was strongly (*P* < 0.001) and the *TMEM106B* variant weakly (*P* < 0.036) associated with LATE-NC.

Dementia was associated with LATE-NC, when all stages were analysed together with Fisher’s exact test (*P* < 0.001), whereas MMSE scores, age at onset and duration of dementia did not reach significance in the combined analysis (Table 2). Table 2 shows the results of association analyses for cognitive parameters when individual stages were separately compared with LATE-NC stage 0 in regression analyses. The LATE-NC stages 1a, 2 and 3 were each significantly associated with dementia (with odds ratios of 3.5, 4.5 and 7.8, respectively, and corresponding *P*-values of 0.01, <0.001 and <0.001, respectively), whereas LATE stages 1b and 1c did not show significant associations. When studying associations between individual LATE-NC stages and MMSE scores (baseline MMSE in 1991 and the last MMSE before death) we found similar results: LATE-NC stages 1a, 2 and 3 were significantly associated with lower MMSE scores, whereas stages 1b and 1c were not (Table 2). Furthermore, LATE-NC stages 2 and 3 were significantly associated with age at onset of dementia and with duration of dementia (*P* < 0.001) (Table 2). The associations of LATE-NC stages 1a, 2 and 3 with dementia and MMSE scores remained significant, when adjusted for ADNC and LRP.

In subsequent analyses, a combination variable including cases with LATE-NC stages 1a, 2 and 3 was used to denote LATE-NC. There were 150 subjects in this LATE-NC group, representing 50.8% of the subjects with LATE-NC staging possible (150/295). This combination variable was compared against the LATE-NC stage 0 group. LATE-NC stages 1b and 1c were excluded from analyses, as they represent minor pathologies, the significance of which is still not established.

### Associations between LATE-NC and other brain pathologies

We next analysed associations between neuropathological variables previously determined in the Vantaa 85+ study and LATE-NC (stages 1a, 2 and 3; seen in Tables 3 and 4). In addition, associations between LATE-NC and arteriolosclerosis variables were assessed (Table 4).

When analysing variables of neurodegenerative pathologies, LATE-NC was most significantly associated with Braak stages V–VI (*P* < 0.001), CERAD score ‘frequent’ (*P* < 0.003), high and intermediate ADNC (*P* < 0.001 and *P* < 0.007, respectively), diffuse neocortical LRP type (*P* < 0.002) and HS (*P* < 0.001). Thal phases 4–5 and Braak stages III–IV were also significantly associated with LATE-NC (*P* < 0.05). In addition, PART was inversely associated with LATE-NC (*P* < 0.001), whereas AGD showed no association with LATE-NC.

**Figure 1:**
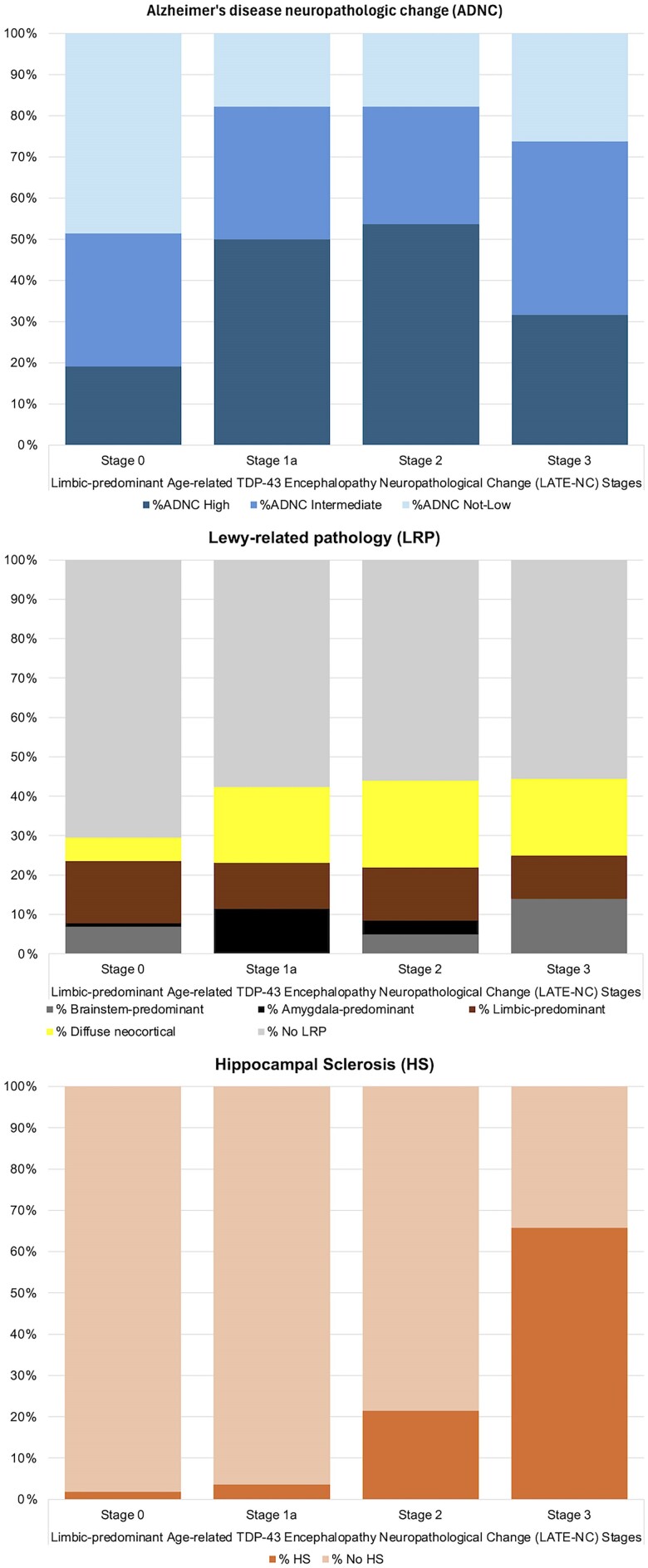
**The frequencies of Alzheimer’s disease neuropathologic change (ADNC), Lewy-related pathology (LRP) and hippocampal sclerosis (HS) co-pathologies in LATE-NC stages 1a, 2 and 3 in the neuropathologically examined Vantaa 85+ subpopulation**. LRP does not include the non-classifiable LRP type (*n* = 11). LATE-NC = limbic-predominant age-related TDP-43 encephalopathy neuropathologic change.

Of the subjects with HS (*n* = 46), all but three cases had either LATE-NC stage 2 or 3 (93.4%). HS was found mostly in subjects with LATE-NC stage 3 (65.8%) and less frequently in subjects with stage 2 (21.4%) (Table 3 and Fig. 1).

Overall, 71.7% of subjects with high ADNC had LATE-NC. Somewhat higher frequencies of ADNC were found in LATE-NC stages 1a and 2, than in stage 3 (Table 3 and Fig. 1).

In total, 73.1% of cases with diffuse neocortical LRP type and 60.0% of subjects with amygdala predominant LRP type showed LATE-NC, whereas the frequencies in the limbic predominant and brainstem predominant LRP types were lower (46.2% and 47.4%, respectively).

We then analysed possible associations between LATE-NC and various vascular changes (Table 4). The strongest association was found with arteriolosclerosis in the amygdala (*P* < 0.011), and we also found an association with arteriolosclerosis in the frontal white matter (*P* < 0.015) but not in the hippocampus. CAA type 1 was nominally significantly associated with LATE -NC (*P* < 0.001), but this association did not remain significant when adjusted for ADNC. Furthermore, infarcts of the posterior circulation were weakly associated with LATE-NC, as were small white matter infarcts and small cerebellar infarcts (*P*-values < 0.05).

It has been reported before that the associations between LATE-NC and neuropathological changes may be different in the oldest old (>90 years) versus younger old (<90 years). Particularly, in the 90+ subjects, LATE-NC has been associated with HS and arteriolosclerosis, but not with ADNC, or LRP.^17^ Therefore, we analysed the associations between LATE-NC and ADNC, HS, LRP, and amygdala arteriolosclerosis in the 90+ subgroup of the Vantaa 85+ neuropathologically examined sample (*n* = 222). In these analyses, the associations between LATE-NC and ADNC, LRP and HS remained significant, whereas arteriolosclerosis was not associated with LATE-NC (Supplementary Table 2).

### LATE-NC versus LATE-NC combined with ADNC and LRP

To assess the interplay of LATE-NC with ADNC and LRP pathologies, we next analysed the cognitive variables and frequencies of common neuropathologies in subjects with different combinations of LATE-NC, ADNC and LRP.

First, we focused on combinations of LATE-NC and ADNC pathology (Table 5). These analyses showed that subjects with LATE-NC combined with ADNC had significantly lower MMSE scores (baseline MMSE *P* < 0.05, last MMSE *P* < 0.01), lower age at onset of dementia (*P* < 0.05) and a longer duration of dementia (*P* < 0.05), compared to subjects with LATE-NC but no ADNC or LRP (designated as LATE-NC only). When comparing these groups, the proportion of subjects with dementia was also higher in subjects with combined ADNC and LATE-NC versus LATE-NC only (80.3% versus 60.0%) but did not reach significance.

As there has been increasing interest in subjects with quadruple misfolded protein phenotypes (subjects with ADNC + LRP + LATE-NC changes) associated with a particularly severe cognitive decline,^35^ we also tested the combination of LATE-NC + ADNC + LRP versus LATE-NC only (Table 5). Subjects with LRP + ADNC + LATE-NC were more often likely to have dementia (93.8% versus 60.0%, *P* < 0.01) and a lower age at onset and longer duration of dementia (*P* < 0.05) than cases with LATE-NC only. There was also a significant difference in MMSE scores (baseline *P* < 0.01 and last MMSE *P* < 0.001) between these groups.

### LATE-NC as the single neurodegenerative pathology leading to dementia

We next took a closer look at subjects with LATE-NC who did not have ADNC or LRP, in order to determine the frequency of subjects with cognitive impairment and LATE-NC, but no other neurodegenerative pathology to explain the cognitive decline. By doing this, we also wanted to see if HS explains the cognitive decline in such cases. We focused on subjects with Braak stage 2 or less, as it is unlikely that this low level of tau pathology would lead to dementia and found 11 cases with LATE-NC, but no ADNC pathology nor LRP (neocortical, limbic, or amygdala predominant) in the Vantaa 85+ cohort. The details of these individuals are shown in Supplementary Table 3.

Six of these 11 subjects were diagnosed with dementia according to DSM-III-R criteria. Of these, five of six subjects had HS, explaining the cognitive decline, whereas only one individual had LATE-NC (stage 2), but did not fulfill the criteria for other common neurodegenerative pathologies, i.e. ADNC, LRP, HS or AGD. When considering vascular pathologies, this individual had a higher than median score for arteriolosclerosis in the frontal white matter, amygdala and hippocampus, and atherosclerosis in the circle of Willis, but no CAA.

### Associations of LATE-NC and other neuropathological variables with dementia in univariate and multivariate models

Finally, we assessed the role of neuropathological changes as predictors of dementia in the Vantaa 85+ population, with a particular focus on LATE-NC’s contribution to dementia in relation to the other common brain pathologies. To do this, we carried out regression analyses with univariate and multivariate models, with dementia as the dependent variable.

The results of univariate models assessing the association of dementia with neurodegenerative and vascular pathologies are shown in Table 6. LATE-NC, ADNC and HS were the neurodegenerative variables most significantly associated with dementia (*P* < 0.001) in univariate models, with LRP also significantly associated with dementia (*P* < 0.004). Of the vascular variables, CAA (*P* < 0.001) and arteriolosclerosis in the amygdala (*P* < 0.002) were most significantly associated with dementia.

The results of various multivariate models are summarized in Table 7. In multivariate analyses, we analysed six different combinations of variables that associated with dementia in univariate models, with the aim of studying the impact of these variables as potentially independent determinants of dementia.

LATE-NC remained an independent, significant predictor of dementia in each model. It is of note that LATE-NC was as strongly, or even more strongly associated with dementia than ADNC in all models. HS had the highest odds ratio (7.4–8.6) in the models where it was included, but it should be noted that there were only a few non-demented subjects with HS and low numbers may have affected the odds ratio of HS. However, according to models 1, 4, 5 and 6, LATE-NC appeared to have an effect independent of HS on dementia in this very elderly population. Associations of HS with other neuropathologies, cognition parameters and risk factors are shown in Supplementary Table 4 (partly previously published in Kero *et al*.^23^).

To estimate the fraction of dementia cases attributable to each dementia-associated pathology, attributable fractions were assessed in each multivariate model. LATE-NC and ADNC showed the highest attributable risk with a magnitude of ∼2 or more times, compared to other dementia-associated pathologies (LRP, HS, arteriolosclerosis) in these models (Table 7). Whenever HS was included in a model, the attributable risk of LATE-NC was lower than of ADNC, possibly reflecting the fact that part of LATE-NC’s impact on dementia is mediated by HS. It is noteworthy that in models 2 and 3, where LATE-NC and ADNC were included without HS, the attributable risk was higher for LATE-NC than ADNC (24.2% versus 18.6% in model 2; 23.0% versus 18.6% in model 3).

## Discussion

Previously, only a few studies have investigated LATE-NC in a population-based setting, i.e. with the sampling frame from a general population defined by geographical boundaries.^4^ Population-based studies are essential for understanding the pathological basis of dementia in a general late-life population for several reasons. These studies allow an unbiased assessment of the prevalence of various brain pathologies, providing information on the full spectrum of diseases and mixtures of pathologies in a population at large, without clinical selection. An additional strength of population cohorts is their longitudinal nature with a rich accumulation of data.^5,36^

Here we used the unique population-based Vantaa 85+ study, which has a high autopsy frequency (>50%) and a wealth of accumulated data, to study the role of LATE-NC in the oldest-old population. Our results provide convincing evidence that LATE-NC is one of the most common forms of neurodegenerative pathologies in the general late-life aged population, and that it is a significant contributor to dementia in the very old age group.

### The frequency of LATE-NC stages

To our knowledge, this is the first study where the recently updated LATE-NC recommendations are applied. Here we report a total frequency of 64.1% for any LATE-NC stage, with frequencies of 22.7% for stage 1 (1a + 1b + 1c), 28.5% for stage 2% and 12.9% for stage 3. These high frequencies highlight LATE-NC as the second most common neurodegenerative pathology after ADNC among the oldest old. Previous studies, based on the criteria published in 2019, have reported frequencies of LATE-NC (all stages combined) varying between 11.1% and 67.7%,^3,4,37-41^ and the following range of frequencies for each stage: stage 1: 4.9%–19%; stage 2: 19%–29%; and stage 3: 2.7%–14%.^3,4,13,37-41^ Thus, the frequencies found in our study are on the higher end of the range reported in previous studies, possibly reflecting the unique nature of our study cohort (see strengths and limitations below). It is of note that comparisons of frequencies between studies are in general complicated by differences in populations, age groups and methods used. Specifically, in the updated criteria,^13^ TDP-43 positive structures are analysed from wider anatomical areas in the hippocampal and amygdala sections than in the first set of criteria published in 2019.^3^ Thus, the updated criteria probably classify more cases with minor pathology as LATE-NC cases, which may have resulted in a higher proportion of stage 1 cases in the present study compared to some previous studies.

### Associations of LATE-NC stages with cognition parameters and risk factors

The LATE-NC stages 1a, 2 and 3 were significantly associated with dementia and MMSE scores (both baseline and last MMSE), with an increasing frequency of dementia and lower MMSE score in each ascending stage. Furthermore, stages 2 and 3 were associated with duration of dementia. Importantly, these associations remained significant when adjusted for two other common neurodegenerative pathologies, ADNC and LRP. The LATE-NC stages 2 and 3 have been shown to be associated with cognition parameters in previous studies,^3,42^ whereas stage 1 has been regarded as an incipient/early-stage pathology,^13^ the impact of which has been uncertain with regards to cognition. In our study, stage 1a showed significant associations with dementia presence and MMSE scores, albeit weaker than stages 2–3, but stages 1b and 1c did not. Our results suggest that NCI of the amygdala region may be the main pathological component of stage 1 associated with cognitive decline, whereas the minor pathologies associated with stages 1b and 1c may represent early-stage pathologies, or even pathologies not directly involved in the process of cognitive decline. A previous study reported a high frequency (40%) of TDP-43 pathology in normal elderly individuals. This pathology consisted mostly of neurites, most commonly found at the uncus of the anterior hippocampus, consistent with LATE-NC stage 1c.^43^

In line with some previous studies, we did not find any significant associations between LATE-NC and use of diabetes or hypertension medication, nor with blood lipid levels (Table 1).^38^ In contrast, a recent study on a 90+ cohort reported that history of hypertension was associated with a reduced likelihood of LATE-NC,^37^ and the ROSMAP study found an association between high haemoglobin 1Ac values and decreased severity of LATE-NC, which remained significant even after controlling for potential confounders.^44^ We report here associations between LATE-NC and *GRN* and *TMEM106B* variants (Table 1), two risk factors that have been previously associated with HS in European populations including the Vantaa 85+ study population.^12,45^ Interestingly, the *GRN* variant was very strongly associated with LATE-NC (*P* < 0.001) in the present study, whereas the association between *TMEM106B* and LATE-NC was weaker (*P* < 0.036). In a recent study, *TMEM106B,* but not *GRN* variant, was associated with LATE-NC, whereas both variants were associated with HS in large datasets of European ancestry.^46^ However, none of these variants were associated with LATE-NC nor with HS in subjects with African ancestries,^46^ consistent with differences in these genetic risk variants in diverse populations. The *APOE ε4* was not an independent risk factor for LATE-NC in the present study, as it lost its significance after adjusting for ADNC. In contrast, some previous studies have reported *APOE ε4* to be associated with LATE-NC^45^ with sensitivity analyses suggesting that this association is independent of ADNC.

### Associations of LATE-NC with other common brain pathologies

We confirm and extend the previous results of strong associations between LATE-NC and ADNC, LRP and HS (Table 3).^3,17,38,39,47-51^ Interestingly, in the present study HS was most commonly found in subjects with LATE-NC stage 3, whereas high ADNC were most common in lower LATE stages (stages 1a and 2) (Table 3 and Fig. 1), indicating possible differences in the pathogenic processes of LATE-NC between these groups. Particularly, the high frequency (65.8%) of HS among LATE-NC stage 3 cases supports the view that HS is the culmination of LATE-NC in subjects with abundant TDP-43 pathology.^52^ In the present study, diffuse neocortical LRP was strongly associated with LATE-NC (Table 3) and confirms the findings by Agrawal and coworkers^53^ in a large community-based sample. This is somewhat different from what was shown in a previous study on the large National Alzheimer’s Coordinating Center (NACC) dataset, where an association between limbic/amygdala-predominant dementia with Lewy bodies (DLB) and LATE-NC was found, but not between LATE-NC and diffuse neocortical or brainstem-predominant DLB.^38^ In agreement with two recent studies,^17,38^ PART was inversely associated with LATE-NC, which may be explained by the strong association between LATE-NC and ADNC, as the definition of PART is not independent of ADNC parameters. AGD was not associated with LATE-NC in the present study, similar to a recent report by Koga and coworkers.^54^ In contrast, some earlier studies have reported an association between TDP-43 pathology and AGD.^43,55,56^

Here we also confirmed and extended some previous reports of associations between LATE-NC and vascular pathologies,^3,9,13,17,38-40,49,57-63^ indicating that dysfunction of the vascular system may have a significant role in the pathogenesis of LATE-NC. In the present study, LATE-NC was associated with arteriolosclerosis in amygdala and in the frontal white matter, but not in hippocampus (Table 4). In previous studies there has been some variation in the location of the arteriolosclerosis associating with LATE-NC. In a study based on the 90+ subpopulation of the Duke Bryan Brain Bank, strong associations between LATE-NC and arteriolosclerosis in the amygdala, hippocampus and frontal cortex were found.^40^ Another study, based on the NACC dataset, reported an association between TDP-43 pathology and arteriolosclerosis in the entorhinal and inferior temporal cortices,^38^ whereas the ROSMAP-MARS study found LATE-NC to be associated with arteriolosclerosis in the posterior watershed area but not the anterior watershed area or basal ganglia.^39^ Interestingly, although arteriolosclerosis in the posterior circulatory regions was not investigated in the present study, we found that cerebral infarcts in the posterior but not anterior circulatory area were associated with LATE-NC (Table 4). Furthermore, the ROSMAP-MARS study reported an association between LATE-NC and type 1 CAA, but not with CAA severity.^39^ In our study, we found a nominally significant association with CAA type 1 (Table 4), but this association was not independent of ADNC pathology, in contrast to the ROSMAP-MARS study.^39^

Our population cohort also allowed us an opportunity to compare subjects with different combinations of the three common neurodegenerative pathologies: ADNC, LRP and LATE-NC. We confirm the findings of several previous studies that the combination of LATE-NC + ADNC leads to more severe cognitive decline than either ADNC or LATE-NC alone (Table 5).^4^ As expected based on previous studies,^35^ a quadruple misfolded protein phenotype showed an even stronger cognitive decline: 93.8% of subjects having ADNC + LRP + LATE-NC had dementia and a median last MMSE score of 0. These results highlight the view that mixed pathology commonly underlies severe cognitive decline in the oldest old population.

A previous report based on two brain bank materials suggested that comorbidities of LATE-NC are different in oldest old versus younger old. Specifically, in 90+ subjects, LATE-NC was associated with HS and arteriolosclerosis, but not with ADNC nor LRP.^17^ However, our population-based study could not confirm these findings (Supplementary Table 2). Further studies on this subject are warranted in other population-representative samples.

### LATE-NC as the single neurodegenerative pathology underlying dementia

We have previously shown that in our cohort of very elderly subjects, virtually all subjects have at least one type of neurodegenerative pathology present in their brains,^19^ and thus it was not unexpected that almost all LATE-NC cases also showed comorbid pathologies. In fact, there was only one individual with dementia (last MMSE score 12) with LATE-NC (stage 2) in our cohort who did not fulfill the criteria for intermediate or high ADNC score, LRP or HS. Even though there was mild tau-pathology present (Braak stage 2), LATE-NC can be interpreted to be the single neurodegenerative pathology as the cause of the severe cognitive decline in this individual. Therefore, it appears that among the oldest old population, it is very rare (frequency < 0.5%) that LATE-NC is the single neurodegenerative disease underlying dementia. In accordance, a recent study based on the 90+ study reported a very low frequency of cases with dementia and LATE-NC (4/364, 1.4%) in the absence of ADNC or LRP among the oldest old.^64^ Interestingly, all these cases had LATE-NC stage 2 and none had HS, similar to our single case. This may imply that these cases might have developed HS, had they lived longer and the LATE-NC progressed to stage 3.

### Multivariate analyses of dementia

One of the greatest strengths of our population-based material is that it provides an opportunity to analyse the full mixture of brain pathologies in combination using multivariate analyses (Table 7). Using this method, we found that LATE-NC is significantly associated with dementia, independently from other major pathologies, thus confirming findings of some previous studies.^17,40,65^ In the previous studies, ADNC showed a higher odds ratio than LATE-NC in multivariate analyses and a greater attributable risk,^3,65^ whilst in our study most models gave a higher odds ratio for LATE-NC than for ADNC, and the attributable risk was also higher for LATE-NC than for ADNC (∼23% versus ∼19%), when these variables were analysed in the same model without HS. Thus, our results, based on an unselected population cohort, suggest that LATE-NC is one of the most significant determinants of dementia in the very aged population and are consistent with some previous studies indicating that the impact of ADNC on dementia at the population level may diminish with increasing age.^1^ Furthermore, our results indicate that LATE-NC has an impact on the development of dementia not just through HS, but also independently from HS, in line with with findings from other studies.^14,45^ In our study, 43/46 subjects with HS had LATE-NC stage 2–3, also supporting the view that HS occurs downstream of LATE-NC.

### Strengths and weaknesses of the study

The main strengths of the study are the well-characterized population-based cohort with a >50% autopsy frequency, a wide and uniform sample collection, and the richness of neuropathological data accumulated since the 1990s (which has also undergone updates with newer detection and scoring methods). All participants represented the genetically homogeneous Finnish population and had reached a very high age. Although they can be regarded as survivors, they have been found to have very high frequencies of different brain pathologies when compared with other studies.^27^ Similarly, the frequency of LATE-NC as reported in the present study was at the higher end of the range reported by previous studies. The birth years of the subjects ranged from 1887 and 1906, and thus life habits were very different from those nowadays. Very few were highly educated. Therefore, our results may not be directly applicable to other populations and younger age groups. For example, the 90+ Study represents mostly highly educated participants, who have lived decades later in a very different environment than the Vantaa 85+ study subjects,^51^ and these differences may account for some discrepancies in results between studies. Although we found very high frequencies of TDP-43 pathology, we cannot exclude the possibility that, due to technical limitations, these frequencies actually represent underestimates. The phosphorylated ser409/410 antibody, which has been used in most LATE-NC studies, did not work optimally in our long-preserved samples and we therefore used the non-phosphorylated antibody, which may be less sensitive.^13^ Furthermore, the amygdala and hippocampal sections used in this study were originally embedded in PEG and later fixed in formaldehyde, whereas the samples from the frontal cortex were originally fixed in formaldehyde. We cannot exclude the possibility that this might have affected the frequency of LATE-NC stage 3 cases in our study. Furthermore, in our study all clinical investigations were performed during the 1990s and cognitive assessments were based only on DSM-III-R criteria for dementia and MMSE tests. This limits clinico-pathological analyses in our study. Finally, neuropathological assessment of the Vantaa 85+ samples is incomplete. For example, at present we have no neuropathological data for age-related tau astrogliopathy (ARTAG) and arteriolosclerosis has only been assessed in three brain areas. Arteriolosclerosis was chosen to be evaluated by SI due to its numerical value, allowing quantitative assessment. However, in this approach, only the thickness of the vessel wall is measured and does not take into account other pathological changes in the vascular wall, nor perivascular alterations, unlike the Vascular Cognitive Impairment Neuropathology Guidelines (VCING).^26,66^

## Conclusion

This population-based study provides evidence that LATE-NC is very common, affecting more than every second very elderly person, and one of the most significant determinants of dementia in the general late-life aged population. However, in the great majority of cases LATE-NC occurs concomitant with other neurodegenerative pathologies and only very rarely (frequency <0.5%) represents the single neurodegenerative pathology underlying dementia.

## Supplementary Material

awae212_Supplementary_Data

## Data Availability

All data used and analysed in this study are available from the corresponding author upon reasonable request.
