# Supplementary material for: Limbic-predominant age-related TDP-43 encephalopathy in the oldest old: a population-based study
Source: Brain. 2024 Jun 28;148(1):154–67. doi: 10.1093/brain/awae212 (PMC11706281; doi:10.1093/brain/awae212)
Supplement: awae212_Supplementary_Data [file awae212_supplementary_data.pdf]

Supplementary material

Limbic-predominant TDP-43 encephalopathy neuropathological change (LATE-NC) is a significant determinant of dementia in the oldest-old - the population-based Vantaa 85+ study

Elizaveta Mikhailenko[1], Kia Colangelo[1], Jarno Tuimala[1], Mia Kero[1, 2], Sara Savola[1, 2], Anna Raunio[1, 2], Eloise H. Kok[1], Maarit Tanskanen[1], Mira Mäkelä[1], Henri Puttonen[1, 2], Mikko I. Mäyränpää[1, 2], Darshan Kumar[3], Karri Kaivola[4, 5], Anders Paetau[1, 2], Pentti J. Tienari[4, 5], Tuomo Polvikoski[6], and Liisa Myllykangas[1,2]

Author affiliations:

[1]Department of Pathology, University of Helsinki, Helsinki, Finland
[2]Department of Pathology, HUS Diagnostic Center, Helsinki University Hospital, Helsinki, Finland
[3] Aiforia Technologies Plc., Helsinki, Finland
[4]Translational Immunology, Research Programs Unit, University of Helsinki, Helsinki, Finland
[5]Department of Neurology, University of Helsinki and Helsinki University Hospital, Helsinki, Finland
[6]Translational and Clinical Research Institute, Newcastle University, Newcastle upon Tyne, United Kingdom

Correspondence to: Liisa Myllykangas, Department of Pathology, University of Helsinki, POB 21, 00014 Helsinki, Finland. Email: liisa.myllykangas@helsinki.fi

Running title: LATE-NC in the oldest-old

Keywords: very old; neuropathology; multivariate analysis; autopsy; neurodegeneration; mixed pathology

Supplementary results

The differential diagnosis between FTLD and LATE-NC can be challenging particularly in cases with abundant TDP-43 pathology in the frontal lobe.[1] In the Vantaa 85+ dataset, most LATE-NC stage 3 cases showed only sparse and focal frontal TDP-43 pathology. However, four cases had relatively abundant frontal TDP-43 pathology, mostly composed of neuritic and punctate pathology and only a few NCI, with the density of TDP-43 positive structures approaching the limit of 15 per high power field, as defined by the updated consensus criteria.[1] Three of these cases exhibited a high or intermediate ADNC score, and one case low. However, none of these four cases fulfilled the criteria for FTLD, as they did not show other characteristic pathological features of FTLD/ALS (such as spongiotic changes in cortical superficial laminae or TDP-43 pathology in hypoglossus or spinal cord regions) and all were clinically diagnosed with Alzheimer's disease type dementia. In addition, each carried either the *GRN (*TT*)* or *TMEM106B* (AA) genotype (associated with LATE-NC), whereas none were found to carry the *C9orf72* expansion or other FTLD-associated gene variants in whole genome sequencing analyses.

Supplementary fig 1

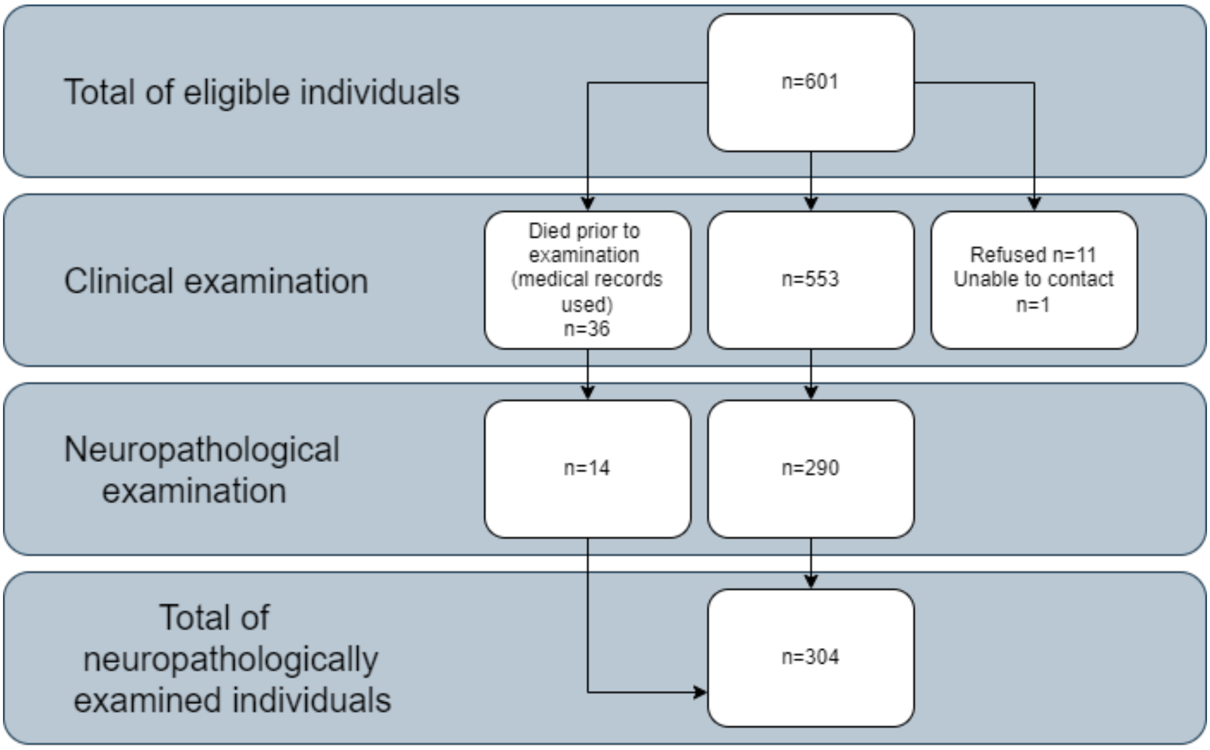

Supplementary fig.1 Flow chart of the Vantaa 85+ study participants

Supplementary fig 2

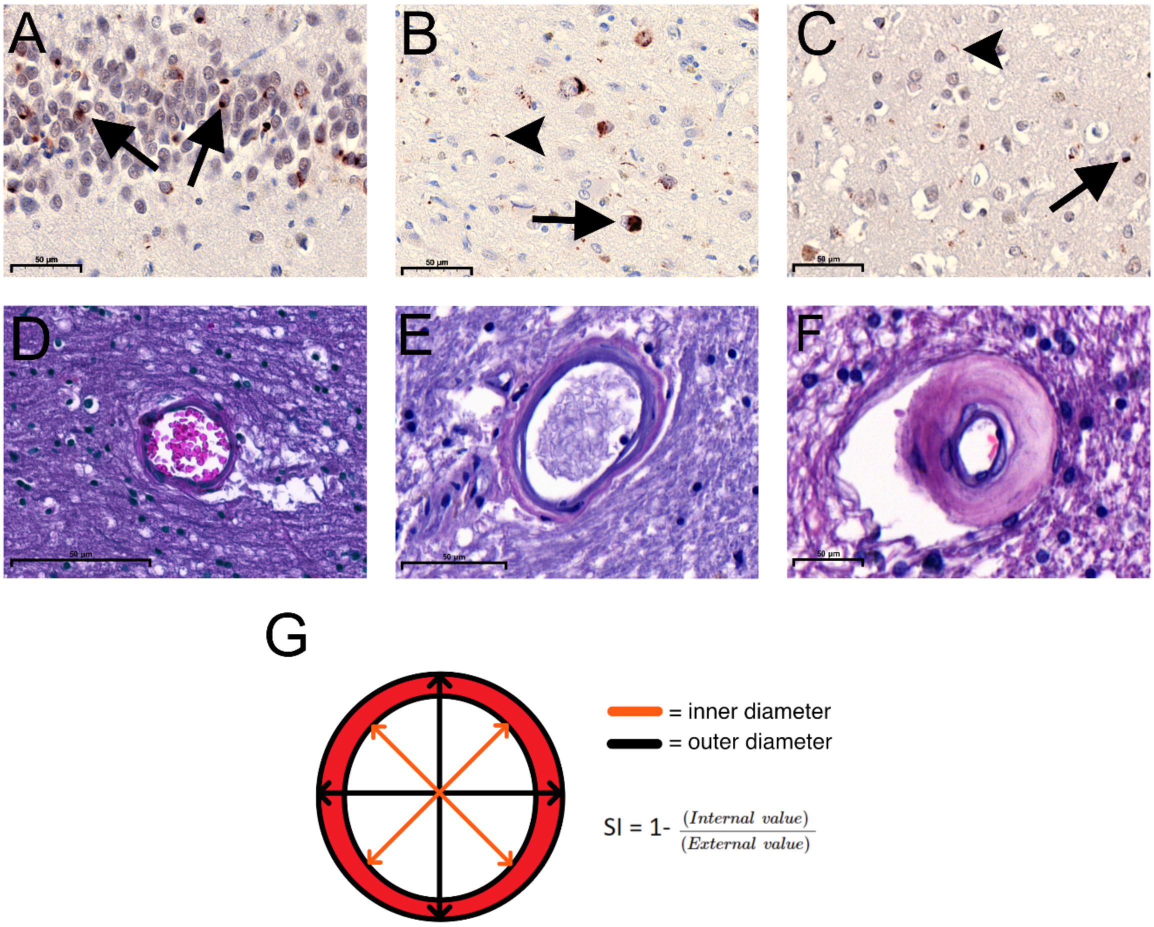

Fig.2 Examples of TAR DNA-binding protein 43 (TDP-43) pathology and arteriolosclerosis. A) Hippocampal dentate fascia granular neurons showing neuronal cytoplasmic TDP-43 positive inclusions. B) Neurons in amygdala showing neuronal cytoplasmic TDP-43 positive (black arrows) inclusions. Black arrowhead indicates a TDP-43 positive process. C) TDP-43 positive neuronal inclusions in frontal cortex (black arrow). Black arrowhead indicates a TDP-43 positive process. D) Normal small arteriole within frontal white matter without arteriolosclerosis. E) Small arteriole with a sclerotic index (SI) of 0.270 in frontal white matter. F) Small arteriole with SI of 0.719 in frontal white matter. G) Formula for calculating SI for each vessel.[2,3]

Supplementary fig 3

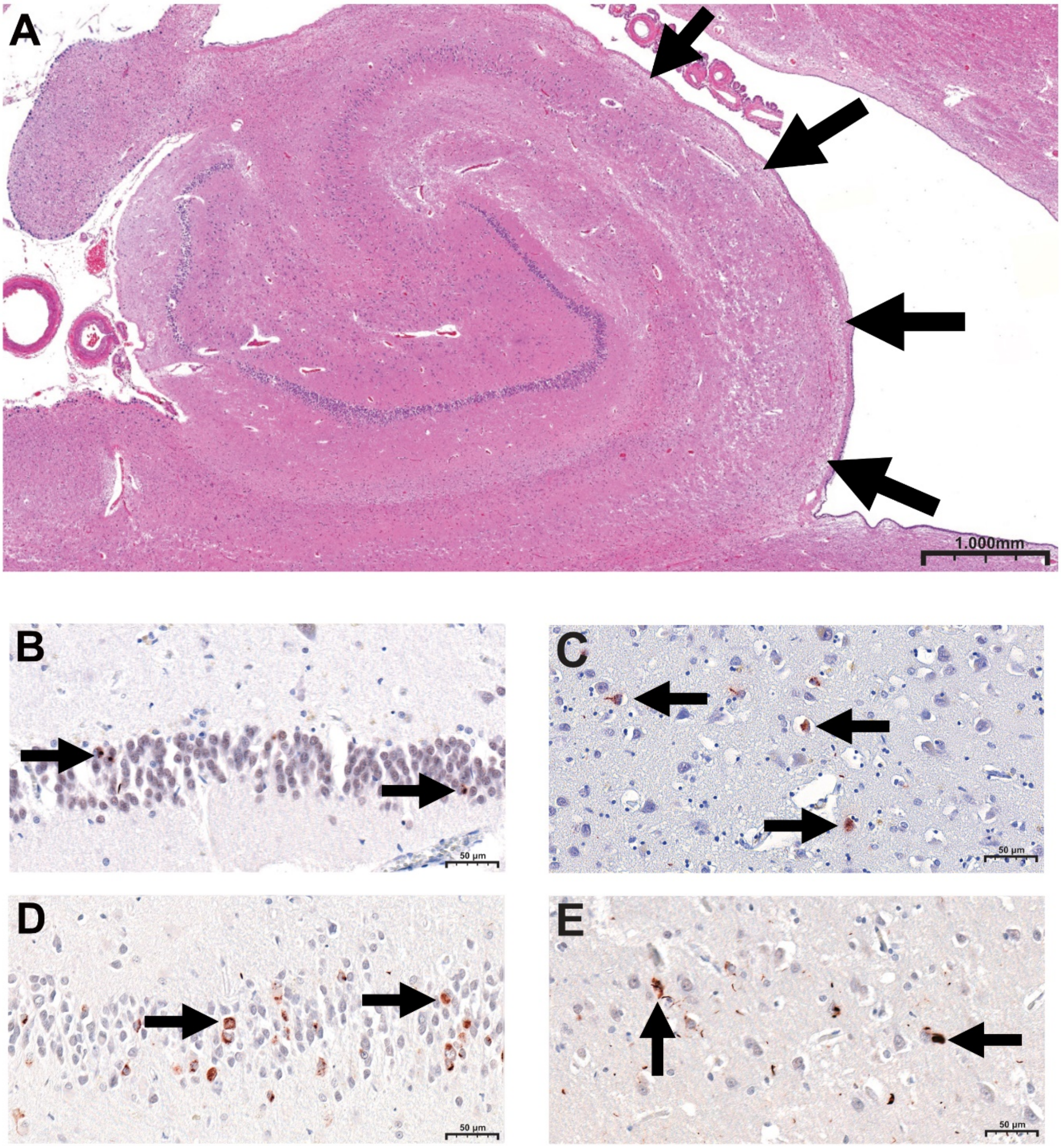

Fig 3 Neuropathological changes in subjects with dementia and LATE-NC, but without high levels of ADNC and LRP pathologies. A) Hippocampal sclerosis seen in a subject with dementia and LATE-NC. Arrows highlight neuronal loss in the CA1 region. In the same individual, neuronal cytoplasmic TDP-43 positive inclusions (arrows) were found in hippocampal dentate fascia granular neurons (B) and amygdala (C). D) The single individual with dementia who did not exhibit ADNC, LRP or HS, showed abundant neuronal cytoplasmic TDP-43 positive inclusions (arrows) in dentate fascia granular cells and E) entorhinal cortex. A) HE staining, scale bar 1mm; B)-E) TDP-43 immunohistochemistry, scale bar 50µm.

Supplementary table 1

Demographic details of the whole Vantaa 85+ study population and of the neuropathologically examined subsample, originally published in Raunio et al., 2019[4], slightly modified version of the table originally published by Oinas et al 2009[5].

	Vantaa 85+ Study population (N=565)	Neuropathological subpopulation (N=304)
Demographic details		
Sex (n, %)		
Men	118 (21)	52 (17)
Women	447 (79)	252 (83)
Age at death (mean ± SD)	91.9 (± 3.6)	92.4 (± 3.7)
Age at death (n, %)		
85-89	188 (33)	82 (27)
90-94	267 (47)	146 (48)
≥ 95	110 (19)	76 (25)
Clinical characteristics		
Dementia status (n, %)		
Dementia	326 (58)	196 (64)
No dementia	239 (42)	108 (36)
Frequency of dementia (n, %)		
Men	63 (53)	30 (58)
Women	263 (59)	166 (66)
Age at onset (mean ± SD)	86.8 (± 4.5)	87.2 (±4.5)
Duration of dementia (mean ± SD)	5.2 (± 3.5)	5.4 (± 3.7)

The study population includes all subjects who died during the ten-year follow-up time and had approved participation in the study.

Supplementary table 2

ADNC, LRP, arteriolosclerosis, and HS by LATE-NC status in participants ≥ 90y

	LATE-NC		
	Stage 0<br>n = 70	Stages 1a or 2 or 3<br>n = 117	*p*
**ADNC, n (%)**			**<0.001**
None-Low	33 (47.1)	22 (18.8)	
Intermediate-High	36 (51.4)	95 (81.2)	
**LRP[a], n (%)**			**0.022**
No	51 (72.9)	65 (55.6)	
Yes	19 (27.1)	52 (44.4)	
**Amygdala arteriolosclerosis[b], n (%)**			NS
None-mild	11 (15.7)	16 (13.7)	
Moderate-Severe	57 (81.4)	97 (83.0)	
**HS, n (%)**			**<0.001***
No	0 (0)	82 (70.1)	
Yes	70 (100.0)	35 (22.9)	

Note: Results of regression analyses with different variables adjusted for age and sex. *CI 95%, P < 0.05 for LATE-NC Stages 1a-2-3
*Calculated using Fischer's exact test because of low numbers.
Abbreviations: ADNC, Alzheimer's disease neuropathological change; LRP, Lewy-related pathology; HS, hippocampal sclerosis; NS, not statistically significant.
[a]All types of LRP included.
[b]We used sclerotic index to distinguish groups: mild when SI < 0.3, moderate 0.3-0.5, severe >0.5. There are no cases without arteriolosclerosis in our cohort.

Supplementary table 3. Cases with LATE-NC but no ADNC* or LRP**

Case nr.	1	2	3	4	5	6	7	8	9	10	11
**Dementia (1/0)**	1	1	1	1	1	1	0	0	0	0	0
**Baseline MMSE score at 1991**	12	17	16	17	21	11	25	24	21	24	25
**The last MMSE score**	12	5	9	13	10	7	22	27	21	24	22
**LATE-NC Stage**	2	3	3	3	2	2	3	2	1a	2	3
**PART (1/0)**	1	0	1	0	0	0	0	1	0	0	0
**Hippocampal Sclerosis (1/0)**	0	1	1	1	1	1	0	0	0	0	0
**CERAD score**	0	1	0	1	2	1	2	0	0	2	1
**Braak NFT Stage**	2	2	2	1	2	2	2	2	2	2	2
**Thal phase**	1	4	2	3	4	5	4	1	3	4	4
**LRP**	0	0	Brainstem predominant	0	0	0	0	0	0	0	0
**AGD (1/0)**	0	0	1	0	0	1	0	1	0	0	1
**CAA Type**	0	Type 2	0	0	0	0	Type 2	Type 2	0	Type 2	Type 2
**Arteriolosclerosis Amygdala**	0.40	0.64	0.49	0.50	0.46	0.45	0.33	#	0.33	0.38	0.66
**Arteriolosclerosis Hippocampus**	0.58	0.50	0.50	0.54	0.59	0.48	0.51	0.43	0.36	0.43	0.53
**Arteriolosclerosis Frontal White Matter**	0.54	0.45	0.48	#	0.59	0.59	0.52	0.46	0.40	0.33	0.61
**Moderate or Severe Atherosclerosis in the Circle of Willis (1/0)**	1	#	1	1	1	1	1	1	1	1	1

*no ADNC and Braak stage less than III.
**no diffuse neocortical, limbic predominant, amygdala-predominant LRP type
#missing value
Abbreviations: ADNC, Alzheimer's disease neuropathological change; AGD, argyrophilic grain disease; CAA, Cerebral amyloid angiopathy; CERAD, Consortium to Establish a Registry for Alzheimer's Disease; LATE-NC, limbic predominant age-related TDP-43 encephalopathy neuropathological change; LRP, Lewy-related pathology; MMSE, Mini-Mental State Examination; PART, primary age-related tauopathy.

Supplementary table 4

Associations between HS and continuous variables by univariable linear regression			Associations between HS and binary variables by univariable logistic regression		
**Variable**	***p***	**Unstandardised B-value for statistically significant* values**	**Variable**	***p***	**OR for statistically significant* values**
Onset of dementia	0.025	-1.78	Dementia (yes/no)	<0.001	15.75
Duration of dementia	<0.001	2.73	ADNC no-low vs intermediate-high score	0.04	2.24
Baseline MMSE score	<0.001	-6.96	LRP (yes/no)	0.92	
Last MMSE score	<0.001	-7.94	AGD (yes/no)	0.82	
SI amygdala	0.11		PART (yes/no)	0.05	0.34
SI hippocampus	0.075		Microinfarcts (yes/no)	0.91	
SI frontal white matter	0.005	0.034	Cortical microhaemorrages (yes/no)	0.33	

Note: Results of association analyses with different variables, not adjusted for age and sex. *$p < 0.05$
Abbreviations: ADNC, Alzheimer's disease neuropathological change; AGD, argyrophilic grain disease; HS, hippocampal sclerosis; LRP, Lewy-related pathology; MMSE, Mini-Mental State Examination; PART, primary age-related tauopathy; SI, Sclerotic index.

References:

1. Nelson PT, Lee EB, Cykowski MD, et al. LATE-NC staging in routine neuropathologic diagnosis: an update. *Acta Neuropathol*. 2023;145(2):159-173. doi:10.1007/s00401-022-02524-2
2. Farber EM, Hines EA, Montgomery H, Craig WM. The Arterioles of the Skin in Essential Hypertension. Published 1947.
3. Lammie GA, Brannan F, Slattery J, Warlow C. Nonhypertensive Cerebral Small-Vessel Disease. An Autopsy study. *Stroke*. 1997;28(11):2222-2229. doi:10.1161/01.str.28.11.2222. PMID: 9368569
4. Raunio A, Kaivola K, Tuimala J, et al. Lewy-related pathology exhibits two anatomically and genetically distinct progression patterns: a population-based study of Finns aged 85+. *Acta Neuropathol*. 2019;138(5):771-782. doi:10.1007/s00401-019-02071-3
5. Oinas M, Polvikoski T, Sulkava R, et al. Neuropathologic Findings of Dementia with Lewy Bodies (DLB) in a Population-based Vantaa 85+ Study. *Journal of Alzheimer's disease*. 2009;18(3).
